# A Preclinical Evaluation of Antimycin A as a Potential Antilung Cancer Stem Cell Agent

**DOI:** 10.1155/2013/910451

**Published:** 2013-06-11

**Authors:** Chi-Tai Yeh, Chun-Li Su, Chi-Ying F. Huang, Justin Kung-Yi Lin, Wei-Hwa Lee, Peter M.-H. Chang, Yu-Lun Kuo, Yu-Wen Liu, Liang-Shun Wang, Chih-Hsiung Wu, Yi-Shing Shieh, Yi-Hua Jan, Yung-Jen Chuang, Michael Hsiao, Alexander T. H. Wu

**Affiliations:** ^1^Graduate Institute of Clinical Medicine, Taipei Medical University, Taipei 11031, Taiwan; ^2^Cancer Center, Taipei Medical University-Shuang Ho Hospital, Taipei 23561, Taiwan; ^3^Graduate Institute of Medical Sciences, National Defense Medical Center, Taipei 11490, Taiwan; ^4^Department of Human Development and Family Studies, National Taiwan Normal University, Taipei 10610, Taiwan; ^5^Institute of Clinical Medicine, National Yang-Ming University, Taipei 11272, Taiwan; ^6^Institute of Biopharmaceutical Sciences, National Yang-Ming University, Taipei 11272, Taiwan; ^7^Cancer Research Center and Genome Research Center, National Yang-Ming University, Taipei 11272, Taiwan; ^8^Department of Chinese Medicine, Taipei Medical University Hospital, Taipei 11031, Taiwan; ^9^Department of Pathology, Shuang Ho Hospital, Taipei Medical University, Taipei 23561, Taiwan; ^10^Division of Hematology and Oncology, Department of Medicine, Taipei Veterans General Hospital, Taipei 11271, Taiwan; ^11^Department of Computer Science and Information Engineering, National Taiwan University, Taipei 10617, Taiwan; ^12^Division of Thoracic Surgery, Department of Surgery, Taipei Medical University-Shuang Ho Hospital, Taipei 23561, Taiwan; ^13^Department of Surgery, Taipei Medical University-Shuang Ho Hospital, Taipei 23561, Taiwan; ^14^Department of Oral Diagnosis, Tri-Service General Hospital, National Defense Medical Center, Taipei 114, Taiwan; ^15^Institute of Bioinformatics and Structural Biology, National Tsing Hua University, Hsinchu 30013, Taiwan; ^16^Genomics Research Center, Academia Sinica, Taipei 115, Taiwan; ^17^The Ph.D. Program for Translational Medicine, College of Medical Science and Technology, Taipei Medical University, 250 Wu-Hsing Street, Taipei 11031, Taiwan; ^18^Translational Research Laboratory, Cancer Center, Taipei Medical University Hospital, Taipei 11031, Taiwan

## Abstract

Drug resistance and tumor recurrence are major obstacles in treating lung cancer patients. Accumulating evidence considers lung cancer stem cells (CSCs) as the major contributor to these clinical challenges. Agents that can target lung CSCs could potentially provide a more effective treatment than traditional chemotherapy. Here, we utilized the side-population (SP) method to isolate lung CSCs from A549 and PC-9 cell lines. Subsequently, a high throughput platform, connectivity maps (CMAPs), was used to identify potential anti-CSC agents. An antibiotic, antimycin A (AMA), was identified as a top candidate. SP A549 cells exhibited an elevated stemness profile, including Nanog, **β**-catenin, Sox2, and CD133, and increased self-renewal ability. AMA treatment was found to suppress **β**-catenin signaling components and tumor sphere formation. Furthermore, AMA treatment decreased the proliferation of gefitinib-resistant PC-9/GR cells and percentage of SP population. AMA demonstrated synergistic suppression of PC-9/GR cell viability when combined with gefitinib. Finally, AMA treatment suppressed tumorigenesis in mice inoculated with A549 SP cells. Collectively, we have identified AMA using CMAP as a novel antilung CSC agent, which acts to downregulate **β**-catenin signaling. The combination of AMA and targeted therapeutic agents could be considered for overcoming drug resistance and relapse in lung cancer patients.

## 1. Introduction

Lung cancer ranks as one of the most deadly malignancies globally and accounts for approximately 0.16 million deaths in the United States alone [1]. Although targeted therapeutic agents such as gefitinib and erlotinib have been shown to be effective in patients with specific mutations in the epidermal growth factor receptor (EGFR) [2, 3], they do not work successfully in EGFR wild-type patients, and even in the patients who are initially sensitive towards these drugs will eventually acquire resistance. For instance, the median progression-free survival after first-line treatment of gefitinib in sensitive EGFR mutated patients is only 10 months [4, 5]. Currently, no alternative and/or effective treatment options can be offered to patients once drug resistance has emerged. Therefore, there is an urgent need to discover and develop alternative agents that can overcome drug resistance in lung cancer patients. Although the mechanism(s) responsible for the development of drug resistance remains elusive, the existence of cancer stem cells (CSCs) provides a rational potential target for investigation. 

CSCs have emerged as one of the hallmarks of cancer and a key contributor to drug resistance and disease relapse. CSCs residing in the heterogenous tumor population are defined based on their ability to seed tumors at limiting dilutions *in vivo*. In addition, CSC-enriched cancer cell populations also exhibit certain properties *in vitro*; thus, CSC-enriched subpopulations can be isolated by utilizing cell-surface marker profiles. For example, leukemia stem cells are enriched for a subset of cells that are CD34^+^/CD38^−^ [6]. Importantly, isolated CSCs can form spherical colonies in suspension cultures that are known as tumor spheroids [7]. Furthermore, CSC-enriched populations exhibit increased resistance to chemotherapeutic agents and ionizing radiation [8]. Collectively, these stem cell-like features define and characterize CSCs. The existence of CSCs has been described in a variety of hematologic and solid malignancies including those of the breast, brain, colon, pancreas, lung, liver, and esophagus [9]. In addition to driving tumorigenesis, CSCs have also been shown to contribute to tumor metastasis and recurrence after treatment. This suggests that current interventions, while killing the bulk of tumor cells, may ultimately fail because they do not eliminate CSCs, which survive to regenerate new tumors. Thus, understanding the underlying mechanisms responsible for the generation of CSCs is essential for developing drugs that may improve treatment efficacy and prevent carcinogenesis of all types of cancer [10]. 

The connectivity maps (CMAP) is a collection of genome-wide transcriptional expression data from cultured human cancer cells that were treated with bioactive small molecules, along with pattern-matching algorithms that collectively facilitate the discovery of connections between queries by the transitory feature of common gene-expression changes. CMAP allow the user to screen compounds against various disease signatures. Drugs are paired with diseases using pattern-matching methods with a high level of resolution and specificity [11]. A strong positive connectivity score (similarity) indicates that a particular drug induces the gene expression of its corresponding query; in contrast, a strong negative connectivity score (dissimilarity) denotes that the agent reverses the expression of the query. Therefore, agents with strong negative connectivity scores could theoretically reverse a particular biological state by modulating the gene signatures. This unique feature of CMAP offers a high throughput platform for pairing potential therapeutic drugs and specific diseases. Taking advantage of this signature pattern-matching feature of CMAP, we hypothesized that if a drug could modulate a set of gene signatures (partially or globally) in CSCs so that the resulting gene pattern resembles the control (parental cell) gene pattern, this particular drug may be effective in controlling or treating CSCs. Using a collection of established gene expression signatures obtained from embryonic stem cells (ESCs) and CSCs as the input for the CMAP database, we have identified antimycin A (AMA), one of the antibiotics in CMAP database, as a potential anti-CSC agent. 

AMA is a potent antifungal agent identified in symbiotic Streptomyces bacteria which reside within the nests of fungal cultivating ants to help confer protection against a range of microfungal weeds [12]. It is also the active component in Fintrol, a chemical piscicide used in fisheries management. AMA functions as a mitochondrial electron transport inhibitor; it has been used to examine the sites of reactive oxygen species generation in mitochondria isolated from chronic obstructive pulmonary disease patients and its relationship with local oxidative stress induced by exercise [13]. Based on its ability to disrupt mitochondrial function, AMA was tested for its potential as an alternative anticancer agent. Recently, AMA has been show to suppress lung cancer cell proliferation by inducing oxidative stress [14]. Interestingly, AMA was identified by CMAP as one of the top-ranking anti-CSC candidates. Here, we intended to examine AMA's potential in suppressing lung CSC generation. First, we isolated and characterized lung CSCs using the side-population method and subsequently examined the effects of AMA on lung CSCs. We found that AMA decreased the self-renewal ability by decreasing their expression of *β*-catenin signaling components. Furthermore, we showed that AMA decreased the percentage of side-population cells in lung cancer cell lines; in combination with gefitinib, AMA effectively eliminated gefitinib-resistant lung cancer cells by negatively modulating *β*-catenin signaling. Finally, AMA was shown to suppress lung tumorigenesis *in vivo*. In summary, we provide evidence that AMA, an antibiotic identified using CMAP, is a potential new agent that can target and eliminate lung CSCs.

## 2. Materials and Methods

### 2.1. Chemicals

Gefitinib was purchased from LC Laboratories (Woburn, MA, USA) and dissolved in dimethyl sulfoxide (DMSO) as a 10 mM stock solution. Antimycin A, Verapamil, Hoechst 33342 and MTT dye (tetrazolium dye (thiazolyl Blue tetrazolium bromide)) was purchased from Sigma-Aldrich (St. Louis, MO, USA). Primary antibodies to *β*-catenin, NF-*κ*B (p65), cyclin D1, TCF-4, Vimentin, and *β*-actin were purchased from Cell Signaling Technology (Boston, MA, USA). CD133/1 (AC133)-PE, human was purchased from Miltenyi Biotec (Cambridge, MA, USA). Antimycin A was dissolved in dimethyl sulfoxide (DMSO) and further diluted in sterile culture medium immediately before use. A TRIzol RNA isolation kit was obtained from Life Technologies (Rockville, MD, USA), and primers for RT-PCR, dNTP, reverse transcriptase, and Taq polymerase were obtained from Gibco BRL (Cergy Pontoise, France). 

### 2.2. Cell Lines and Culture

Lung cancer cell lines A549 and PC-9 were obtained from the ATCC. PC-9 is derived from a patient with adenocarcinoma and harbors an EGFR exon 19 in-frame deletion that is highly sensitive to EGFR-TKIs. PC-9/GR is a cell line resistant to gefitinib that was established by chronic exposure of PC-9 cells to medium containing increasing concentrations of the drug [15]. Briefly, PC-9 cells were first exposed to 10 nmol/L of gefitinib in medium containing 10% fetal bovine serum, and the concentration of gefitinib was then increased in a stepwise manner. Cells that were able to grow in 1 *μ*mol/L gefitinib were obtained 6 months after initial expose. The decreased sensitivity did not reverse even after the cells were kept in culture for >4 months without gefitinib. These cells were grown in RPMI 1640 culture medium or Dulbecco's modified Eagle's medium (GIBCO-Life Technologies, Inc., Gaithersburg, MD) supplemented with 10% fetal bovine serum (FBS), penicillin (100 UI/mL), and streptomycin (100 *μ*g/mL) at 37°C in a humidified atmosphere with 5% CO_2_ and harvested with trypsin-EDTA when the cells were in exponential growth.

### 2.3. Isolation of A549 Side Population Cells by Cell Sorting

To examine the existence of CSCs in an established A549 carcinoma cell line, side population (SP) cells were isolated by flow cytometry and cell sorting techniques. SP cells that expressed ATP-binding cassette (ABC) transporters (ABCG2) and Hoechst 33342 efflux activity were sorted by FACSaria flow cytometry. Lung cancer cells were labeled with 2.5 *μ*g/mL Hoechst 33342 (Sigma-Aldrich) for 30 min at 37°C. The control cells were incubated in the presence of 25 *μ*M verapamil (Sigma-Aldrich), a broad spectrum ABC transporter inhibitor. Propidium iodine (PI) (1 *μ*g/mL) was added to discriminate dead cells. Analysis and sorting were performed on FACSaria flow cytometers (Becton Dickinson, San Jose, CA). After sorting, A549 SP sphere cells were plated at a density of 1000 cells/mL under stem cell conditions by resuspending them in tumor sphere medium consisting of serum-free HEScGRO medium, N2 supplement (Invitrogen, Carlsbad, CA), 10 ng/mL human recombinant bFGF (Invitrogen, Calsbad, CA), and 10 ng/mL EGF; subsequently, they were cultured in ultralow attachment plates (Corning, NY, USA) for approximately 1 week.

### 2.4. Assessment of the Growth of A549 SP and NSP Cells following Antimycin A Treatment

Sulforhodamine B (SRB) dye (Sigma-Aldrich, Munich, Germany) was used to test the effects of selective inhibitors on cell growth and viability of SP cells. Antimycin A was dissolved in DMSO before diluting with growth medium to a final DMSO concentration of <0.05%. The A549 SP cells were seeded into 96-well plates in growth medium at 500 cells/well. After 24 h, the medium was replaced with fresh growth medium containing antimycin A, and the cells were incubated for another 48 h. The cells were then fixed by gently adding 50 *μ*L TCA (50%) to each well for a final TCA concentration of 10%, with subsequent incubation for 1 h at 4°C. The plates were then washed 5 times with tap water and air dried. The dried plates were stained with 100 *μ*L of 0.4% (w/v) SRB prepared in 1% (v/v) acetic acid for 10 min at room temperature. The plates were rinsed quickly 4 times with 1% acetic acid to remove unbound dye and then air dried until no moisture was visible. The bound dye was solubilized in 20 mmol/L Tris base (100 *μ*L/well) for 5 min on a shaker. Optical densities were read on a microplate reader (Molecular Devices, Sunnyvale, CA) at 562 nm.

### 2.5. *β*-Catenin/TCF Transcription Reporter Assay

A549 SP cells were plated in 6-well plates, grown to 80%–90% confluence, and transiently transfected with TOPflash and FOPflash plasmids, respectively. TOPflash has 3 copies of the Tcf/Lef binding sites upstream of a thymidine kinase (TK) promoter and the firefly luciferase gene. FOPflash is used as control for measuring nonspecific activation of the reporter. All transfections were performed with Lipofectamine and 0.3 *μ*g of TOPflash or FOPflash plasmids. To normalize transfection efficiency, cells were cotransfected with 0.2 *μ*g of the internal control reporter encoding Renilla reniformis luciferase driven under the TK promoter. After transfection, cells were incubated in medium with or without antimycin A (0–10 *μ*M) for 48 hours and then lysed with reporter lysis buffer. Luciferase activity was determined by Dual-Luciferase Assay System kit according to vendor's protocol. The experiments were performed in triplicate, and the results were reported as folds of induction compared with control group after normalization to transfection efficiency.

### 2.6. RT-PCR

Total RNA was purified from the SP cells using Trizol reagent (Invitrogen, Carlsbad, CA) according to the manufacturer's protocol. RNA (2 *μ*g) was added to RT-PCR reactions (final primer concentration of 0.5 *μ*M). After a 42°C/60 min reverse transcription step, 30 cycles of amplification were performed at 94°C for 30 sec, 58°C for 50 sec, and 72°C for 50 sec. PCR products were run on 1.5% agarose gels for identification. Primers used were 5′-CAG AGT ACA ACG CCA AAC CA-3′ and 5′-AAA TCA CGA TGA GGG TCA GC-3′ for CD133, and 5′-GAA GGT GAA GGT CGG AGT C-3′ and 5′-CAA AGT TGT CAT GGA TGA CC-3′ for GAPDH. The Q-PCR primers used for detecting gene expression of CD133, Sox2, *β*-catenin, and Nanog were as follows: CD133-specific primers (sense primer 5′-TCTTGACCGACTGAGACCCAAC-3′ and antisense primer 5′-ACTTGATGGATGCACCAAGCAC-3′), Sox2-specific primers (sense primer 5′-GACAGTTACGCGCACATGAA-3′ and antisense primer 5′-TAGGTCTGCGAGCTGGTCAT-3′), *β*-catenin-specific primers (sense primer 5′-GCGTGGACAATGGCTACTCAAG-3′ and antisense primer 5′-TATTAACTACCACCTGGTCCTC-3′), and Nanog-specific primers (sense primer 5′-GTGATTTGTGGGCCTGAAGA-3′ and antisense primer 5′-ACACAGCTGGGTGGAAGAGA-3′).

### 2.7. Western Blotting

Cell lysates were prepared using a ReadyPrep Protein Extraction Kit (Bio-Rad, Hercules, CA) according to instructions provided. Total cell lysates (50 *μ*g) were separated using 10% SDS-PAGE and transferred onto a polyvinylidene fluoride membrane using the BioRad Mini Protean transfer system. The blots were then blocked with 5% skim milk in PBST for 1 h and probed with primary antibodies overnight at 4°C. The membrane was further incubated overnight at 4°C with respective specific antibodies against active *β*-catenin (1 : 1000), TCF-4 (1 : 1000), cyclin D1 (1 : 1000), NF-*κ*B/p65 (1 : 1000), and *β*-actin (1 : 5000). After incubation with primary antibodies, the membrane was washed with TBST 3 times. The membrane was then incubated with horseradish peroxidase-labeled secondary antibody for 45 minutes at room temperature and washed with TBST 3 times. Final detection was performed with enhanced chemiluminescence (ECL) Western blotting reagents and the BioSpectrum Imaging System (UVP, Upland, CA).

### 2.8. *In Vivo* Evaluation of the Effects of Antimycin A on Cancer Stem Cells

All animal studies were performed strictly under the animal experimentation protocols approved by Taipei Medical University. A549 side-population cells were first modified to express the dual reporter system FUW-Luc-mCherry-Puro (a generous gift from Dr. Andrew Kung, Lurie family Imaging Center, Dana Farber Cancer Institute, MA). Imaging-ready A549 side-population cells were harvested and injected via the tail vein of NOD/SCID mice (5.5 × 10^5^ cells). Tumor-bearing mice were then subdivided into control and Antimycin A-treated groups (10 mg/kg i.p. injection, 3 times a week). Tumor burden was noninvasively assessed based on bioluminescence intensity for 4-5 weeks using the IVIS200 system (Caliper life sciences Inc., Hopkinton, MA). Tumor biopsies were obtained at the end of the experiment by humanely sacrificed the animals.

### 2.9. Histology and Immunohistochemical Staining

Tumor tissues were fixed in 10% formalin and embedded in paraffin. Serial sections of the embedded specimens were deparaffinized and then rehydrated in a graduated fashion and stained with hematoxylin and eosin (H&E). For immunohistochemical staining, the deparaffinized slides were subjected to antigen retrieval and probed with anti-beta-catenin (1 : 100), anti-NF-*κ*B-p65 (1 : 200), anti-Vimentin (1 : 100), or anti-E-cadherin (1 : 100) antibodies, or isotype IgG control. Slides were washed and incubated with biotinylated link universal antiserum, followed by horseradish peroxidase-streptavidin conjugate (LSAB 1 kit). The slides were rinsed, and color was developed using 3,3-diaminobenzidine hydrochloride as a chromogen. Finally, sections were rinsed in distilled water, counterstained with Mayer's hematoxylin, and mounted with DPX mounting medium for evaluation. Pictures were captured with a Photometrics CoolSnap CF color camera (Nikon, Lewisville, TX).

### 2.10. Statistical Analysis

Each experiment was performed in triplicate. The results were expressed as the means ± SEM. The comparisons of means between two groups were analyzed with independent sample *t*-tests. For comparison of more than two groups, Analysis of Variance (ANOVA) was conducted to examine the equality of means. A Bonferroni post hoc analysis was executed following a significant ANOVA to detect differences. The threshold of significance was set at *P* = 0.05 throughout the study.

## 3. Results

### 3.1. Identification of Antimycin A (AMA) as a Potential Anti-CSC Agent Using the Connectivity Map Database

**Figure 1:**
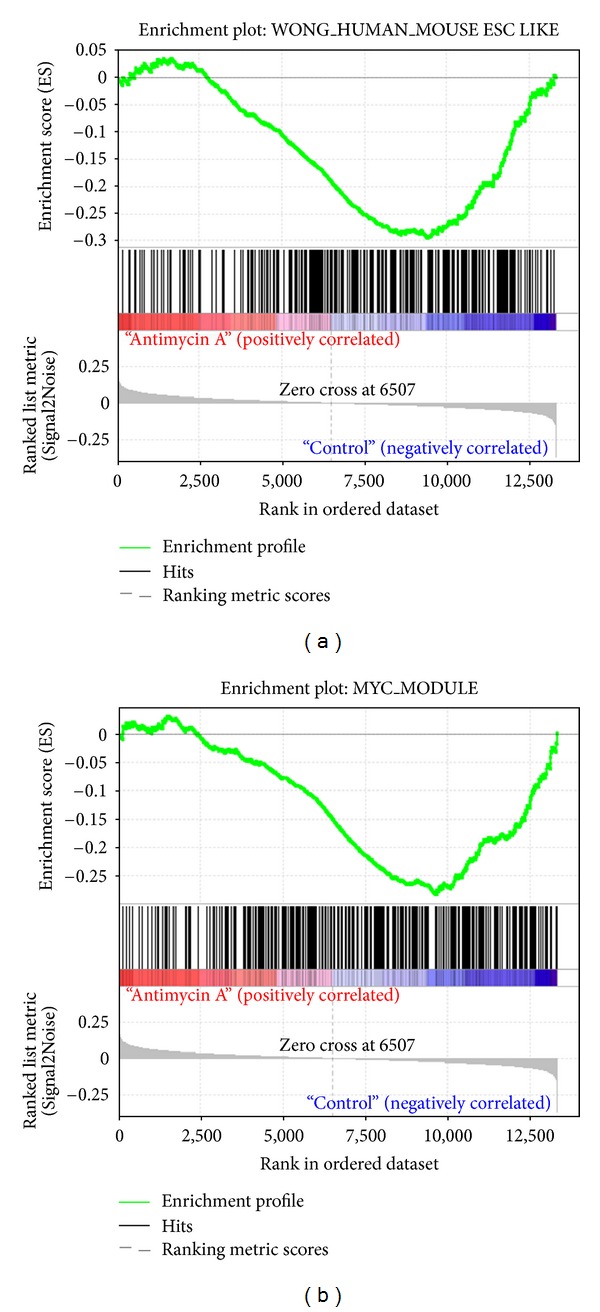
Identification of antimycin A as a potential anti-CSC agent using the connectivity maps database. Gene set enrichment analysis (GSEA) demonstrated that the AMA drug signature reverses Wong's ESC module (a) and the Myc module from Kim's study (b). Both modules have been correlated with CSC-like phenotypes, suggesting antimycin A have the potential to reverse lung CSC signature.

Using a CMAP algorithm in combination with gene signatures from ESCs and CSCs, we were able to identify a group of antibiotics from the CMAP database that have the potential to reverse the CSC-associated gene signatures (see Supplementary Table  1 available online at http://dx.doi.org/10.1155/2013/910451). One of the top-ranking candidates was AMA. A previous study showed the ESC transcription program used by Wong and coworkers [16] as similar to the Myc module [17]. Therefore, AMA signatures obtained from CMAP were subsequently subjected to Gene Set Enrichment Analysis (GSEA), which is a computational method that determines whether an a priori defined set of genes shows statistically significant, concordant differences between two biological states. The concordant gene expression behavior of the AMA signature was found to reverse both ESC and Myc modules, which are very close to each other and correlate well with CSC-like phenotypes (Figure 1). This analysis raised the possibility that targeting these specific cancer-associated ESC-like gene signatures could result in the inhibition of CSCs. In addition, treatment of lung cancer stem cells (CL141) with AMA resulted in downregulation of c-Myc (data not shown), suggesting that AMA has the potential to reverse lung CSC-like gene signatures. 

### 3.2. Identification and Characterization of Side-Population Cells from the Lung Cancer Cell Line A549

**Figure 2:**
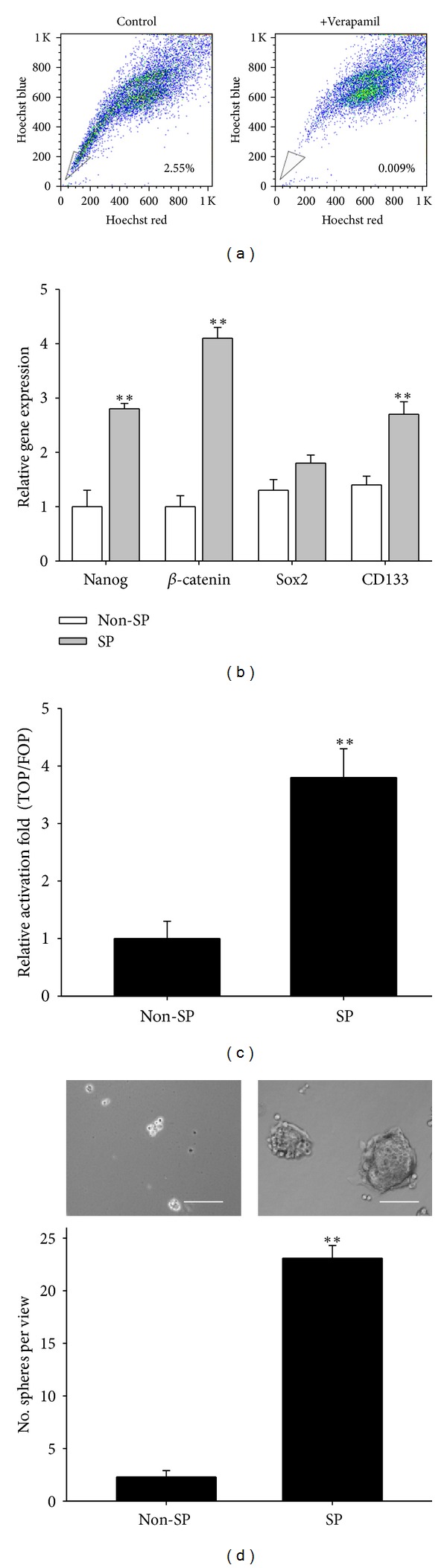
Characterization of lung cancer stem cell properties via side population assays. (a) Side-population flow cytometric analysis of A549 lung cancer cells. Approximately 2.55% of cells were identified as SP. (b) Real-time PCR analysis showed that A549 SP cells contained higher levels of Nanog, *β*-catenin, Sox2, and CD133 mRNA transcripts compared to their non-SP counterparts. (c) A549 SP cells showed higher *β*-catenin-TCF/LEF transcriptional activity than their non-SP counterparts. TOPFLASH (TOP) or FOPFLASH (FOP) plasmids (0.3 *μ*g) were used for the reporter assay. (d) A549 SP cells showed a higher ability (approximately 8-fold) to form tumor spheroids in serum-deprived medium culture than non-SP cells. Scale bar, 50 *μ*m. *denotes *P* < 0.05 while **represents *P* < 0.01 as compared to their respective controls.

To validate the potential anti-CSC function of AMA, a consistent and reliable cell model of lung CSCs was required. Based on this premise, we first identified and isolated SP cells from the A549 lung cancer cell line by flow cytometry based on the SP's ability to exclude Hoechst 33342 DNA binding dye (Figure 2(a)). The isolated SP cells demonstrated a marked elevation of stem cell-associated mRNA transcripts, including Nanog, *β*-catenin, Sox2, and CD133, compared to the non-SP counterparts (Figure 2(b)). In addition, the transcriptional activity of *β*-catenin-TCF/LEF was markedly increased in SP cells compared to non-SP counterparts (Figure 2(c)). More importantly, when cultured in serum-deprived stem cell medium, SP cells demonstrated an enhanced ability to form tumor aggregates or spheroids, while non-SP cells showed a significantly lower ability to form tumor spheroids (Figure 2(d)). Collectively, SP cells isolated from the A549 lung cancer cell line demonstrated a spectrum of stem cell-like characteristics.

### 3.3. Antimycin A Suppresses the Ability of A549 SP Cells to Form Tumor Spheroids by Disrupting *β*-Catenin/TCF-4 Signaling

**Figure 3:**
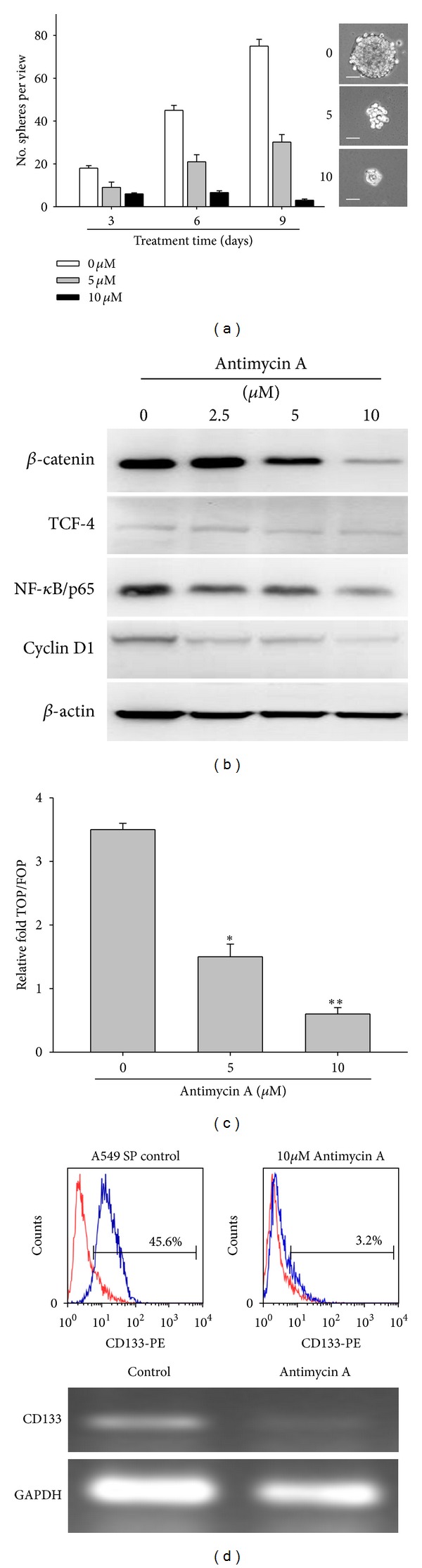
Antimycin A dose dependently suppresses the self-renewing ability of lung cancer stem cells. (a) Different concentrations of AMA (0, 5, and 10 *μ*M) were used to treat A549 tumor spheroids. AMA exhibited a dose-dependent ability to inhibit tumor sphere formation. Inserts depict the morphology of AMA-treated tumor spheroids. Scale bar, 50 *μ*m. (b) Western blot analysis of AMA-treated A549 tumor spheroids. AMA dose dependently suppressed the expression of components of the *β*-catenin signaling cascade. (c) TOP/FOP luciferase assay was used to examine AMA's ability to suppress *β*-catenin-TCF/LEF transcriptional activity in A549 SP cells. AMA treatment significantly suppressed the transcriptional activity of the *β*-catenin pathway as indicated by decreased luciferase activity as the concentration of AMA increased. (d) Flow cytometric and RT-PCR analyses of AMA-mediated downregulation of CD133 expression. By flow cytometric analysis, AMA treatment suppressed the percentage of A549 SP cells that expressed CD133 by approximately 40%. Consistently, RT-PCR analysis demonstrated a significantly downregulated CD133 transcript in AMA-treated A549 SP cells. GAPDH served as internal control. *denotes *P* < 0.05 while **represents *P* < 0.01 as compared to their respective controls.

Once establishing A549 SP cells as tumor cancer stem-like cells, we subsequently used this platform to examine AMA's ability to suppress the stemness of A549 SP cells. AMA treatment suppressed the tumor spheroid-forming ability of A549 SP cells in a dose-dependent manner (Figure 3(a)). Because *β*-catenin/TCF-4 signalling plays a pivotal role in stem cell development [18, 19], we examined whether AMA's ability to suppress the formation of tumor spheroids was through this pathway. AMA treatment significantly downregulated the expression of *β*-catenin/TCF-4 and cyclin D1 in A549 SP cells at a low concentration (2.5 *μ*M). At higher concentrations (5–10 *μ*M), AMA suppressed the expression of two major signalling pathways that are often dysregulated in cancer cells, namely, Wnt/*β*-catenin and NF-*κ*B (Figure 3(b)). Additionally, AMA inhibited *β*-catenin transcription in A549 SP cells (Figure 3(c)). Of equal importance, AMA-treated A549 SP cells exhibited decreased CD133 expression (from 45.6% in control cells to 3.2% in treated cells) as demonstrated by flow cytometry (upper panels, Figure 3(d)) and RT-PCR analysis (lower panels, Figure 3(d)). 

### 3.4. Antimycin A Targets the Gefitinib-Resistant Lung Cancer Cell Line PC-9/GR

**Figure 4:**
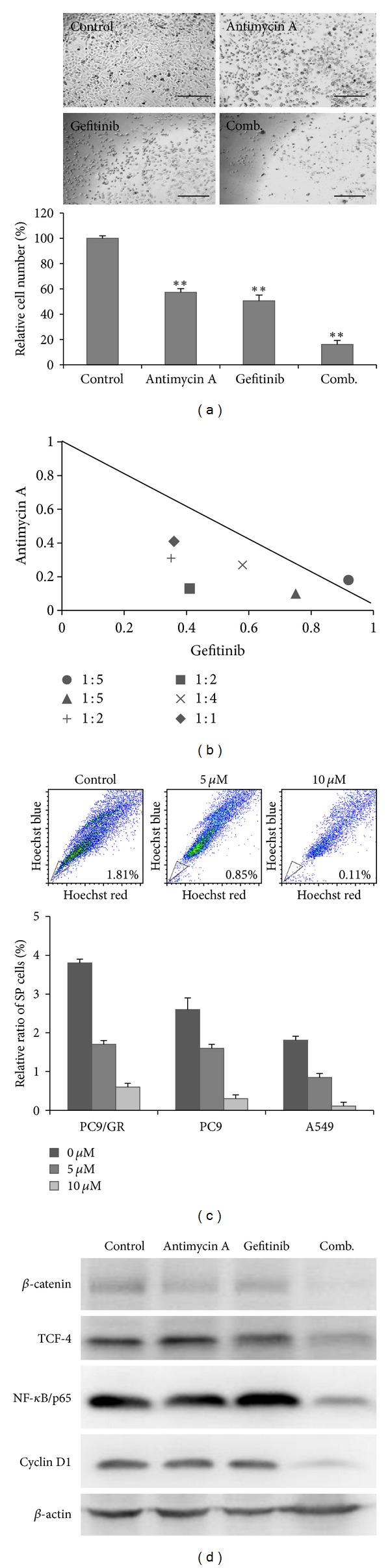
Antimycin A overcomes gefitinib resistance in lung cancer cells. (a) Gefitinib-resistant PC-9 lung cancer cells (PC-9/GR) were treated with gefitinib (5 *μ*M), AMA (5 *μ*M), and the two agents combined. AMA or gefitinib alone could only suppress PC-9/GR proliferation by approximately 35% and 42%, respectively. Gefitinib and AMA combined synergistically suppressed PC-9/GR proliferation by approximately 80%. Scale bar, 100 *μ*m. (b) Isobologram analysis showed that the combination of gefitinib and AMA suppressed PC-9/GR proliferation synergistically. Normalized isobolograms are shown where PC-9/GR were exposed for 48 h to different combinations of AMA (0.5, 2.5, and 5 *μ*M) and gefitinib (2.5, 5, and 10 *μ*M). Symbols designate the combination index value for each fraction affected. The curves were generated by Calcusyn software to fit the experimental points. The data are representative of three independent experiments. Values below the line are synergistic, whereas those close to the line are additive and those above the line antagonistic. (c) AMA dose dependently decreased the percentage of side-population cells in lung cancer cells. A representative flow cytometric analysis showed that AMA decreased the percentage of SP cells in the A549 cell line (upper panel); AMA also suppressed the SP cell populations in PC-9 and PC-9/GR cell lines (bottom panel). (d) Western blot analysis demonstrated that gefitinib (5 *μ*M) and AMA (5 *μ*M) combination treatment significantly inhibited *β*-catenin-associated signaling components in A549 SP cells. *denotes *P* < 0.05 while **represents *P* < 0.01 as compared to their respective controls.

Acquired resistance towards targeted therapeutic agents represents one of the major properties of cancer stem cells. Thus, we intended to test if AMA could target gefitinib-resistant lung cancer cells. PC-9/GR cells were grown in the presence of gefitinib, AMA, or both drugs (Figure 4(a)). AMA treatment (5 *μ*M) inhibited the proliferation of PC-9/GR cells to the smallest extent (35%), followed by gefitinib treatment (42%). Importantly, the combination of both drugs eradicated approximately 80% of PC-9/GR cells. Isobologram analysis indicated that AMA and gefitinib worked synergistically to eliminate PC-9/GR lung cancer cells (Figure 4(b)). 

One of the key features of side-population cells is their high expression of ABCG2 pumps and the ability to exclude Hoechst dye (reflecting their ability to exclude drugs). Thus, to demonstrate AMA's ability to overcome drug resistance, PC-9/GR (gefitinib-resistant PC-9) cells were subjected to SP analysis. When treated with AMA (5 and 10 *μ*M), the percentage of A549 SP cells (1.81% in control samples) decreased to 0.85% and 0.11%, respectively (upper panels, Figure 4(c)). Similarly, SP cells from PC-9 (wild-type) and PC-9/GR cells were also examined. We found that the initial percentage of SP cells in each cell line was approximately 2.6% and 3.8%, respectively (lower panels, Figure 4(c)). Again, AMA treatment eliminated SP cells in a dose-dependent fashion. To explore the molecular mechanisms mediated by AMA, total protein lysates were harvested from A549 SP cells. The expression levels of stemness molecules including *β*-catenin and TCF4 were decreased. NF-*κ*B/p65 and cyclin D1 expression was also suppressed by AMA and the combined treatment (Figure 4(d)). These data demonstrate that AMA possesses the ability to eliminate cancer stem-like SP cells in drug resistant lung cancer cells, both alone and in combination with other treatments. 

### 3.5. Antimycin A Inhibits Tumorigenesis *In Vivo*

**Figure 5:**
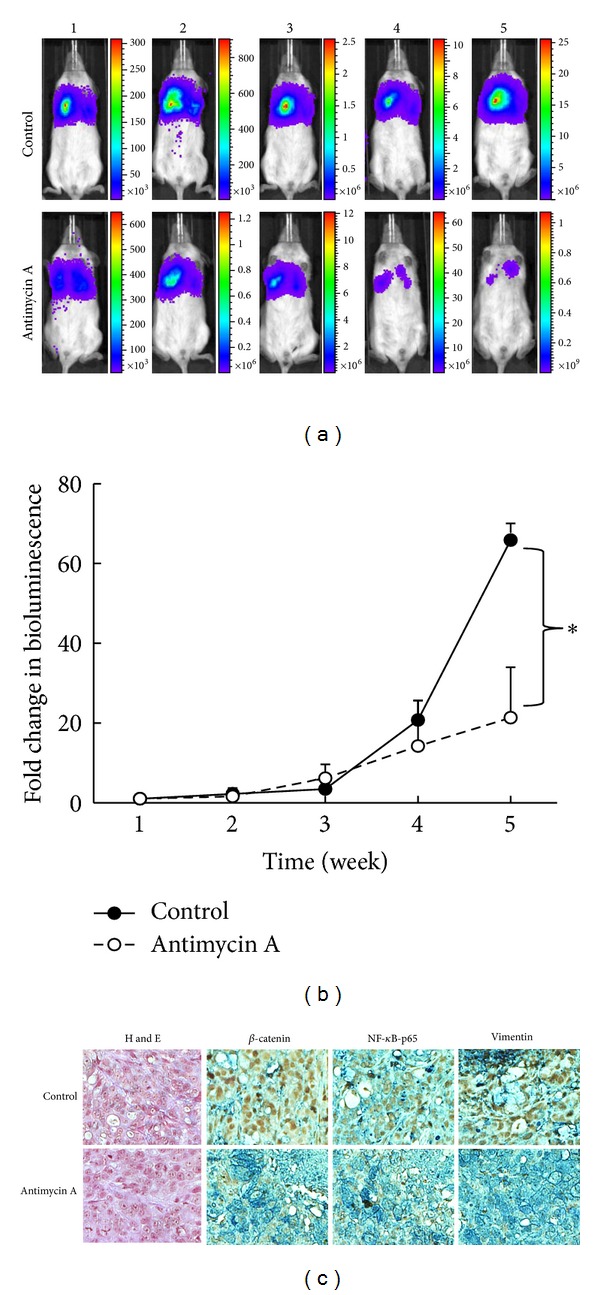
Antimycin A suppresses lung tumorigenesis *in vivo*. (a) Noninvasive bioluminescence imaging was used to monitor the tumor inhibitory effects of AMA *in vivo*. Mice bearing A549 SP cells were divided into control and AMA groups (*N* = 5 each group). Over time, AMA-treated mice demonstrated suppressed lung tumorigenesis, as demonstrated by lower bioluminescence signals. (b) Quantitative bioluminescence data demonstrate the fold changes in bioluminescence signal over time from both control and AMA groups. (c) Immunohistochemical analysis of tumor biopsies. AMA-treated sections demonstrated weaker staining of *β*-catenin and NF-*κ*B-p65 compared to those in the sections from control animals. Analysis of EMT markers indicates that AMA-treated sections showed decreased Vimentin and increased E-cadherin as compared to the sections obtained from control mice. *refers to the significant difference observed in the fold change in bioluminescence between control and antimycin A groups. *P < 0.05.

Tumor-initiating ability is one of the most important hallmarks of cancer stem cells. Therefore, we further explored the anticancer stem cell ability of AMA using an immune compromised mouse xenograft model. Firefly luciferase-expressing A549 SP cells were subcutaneously injected into NOD/SCID mice; mice were then divided into two groups: one receiving AMA treatment (i.p. injection, 10 mg/kg, 3 times/week) and one receiving vehicle. Tumorigenesis was monitored using a noninvasive molecular system. Three weeks after inoculation, AMA-treated mice exhibited a lower ability to initiate lung tumors (lower panel, Figure 5(a)) when compared to the vehicle-treated group (upper panel, Figure 5(a)) as reflected by the bioluminescence images. The tumor burden was monitored using the change in bioluminescence over a period of 5 weeks (after tumor inoculation), and the AMA-treated group demonstrated a significantly lower increase in bioluminescence as compared to the vehicle-treated counterpart. The data from bioluminescence imaging was supported by immunohistochemical analysis of the tumor biopsies obtained from both groups. Tumor sections from AMA-treated mice demonstrated a decreased expression of *β*-catenin, NF-*κ*B/p65, and Vimentin, and an increased expression of E-cadherin (lower panels, Figure 5(c)), compared to samples from the control mice (upper panels, Figure 5(c)). Collectively, these findings suggested that AMA treatment efficiently suppressed tumorigenesis *in vivo* by negatively modulating *β*-catenin (stemness marker), NF-*κ*B (inflammatory marker), and Vimentin (mesenchymal marker). 

## 4. Discussion

Drug resistance, metastasis, and disease recurrence have been the major obstacles encountered in the management of cancer patients. Lung cancer remains a major cause of cancer-related lethality due to high incidence and recurrence in spite of significant advances in staging and therapies [20, 21]. Studies have demonstrated that stem cells present in the airways may be the initiators of lung tumorigenesis. These putative stem cells exhibit tumorigenic characteristics, including a high proliferative ability, multipotent differentiation, drug resistance, and increased metastatic potential compared to other cells [22, 23]. Therefore, these so-called lung CSCs represent a target for drug development. To test our hypothesis, we used a CMAP database in combination with gene signatures from ESCs or CSCs and identified AMA as a potential anti-CSC agent. Of equal importance, we utilized flow cytometry (side-population) to identify and isolate lung CSCs for evaluating the anti-CSC functionality of AMA. AMA was shown to significantly suppress the self-renewing ability of A549 CSCs by negatively modulating the *β*-catenin signaling cascade. More importantly, using the gefitinib-resistant lung cancer cell line PC-9/GR, we showed that AMA overcame gefitinib resistance. Finally, AMA's antitumor ability was validated *in vivo*. 

The CMAP system was originally developed to generate a detailed map that links gene patterns associated with disease to corresponding patterns produced by drug candidates and a variety of genetic manipulations. We took advantage of this system by incorporating ESC or CSC signatures into the algorithm. The reason for evaluating the ESC signatures is that certain phenotypes are found to be crossover between ESCs and CSCs [16, 17]. Specifically, we searched for drugs in the database which were capable of “reversing” the ESC signature to one which resembles to a normal non-CSC signature. From the GSEA analysis, it is interesting to find that AMA reverses both ESC modules from the Wong et al. and Kim et al. studies [16, 17], which have correlated well with CSC-like phenotypes such as relapse and progression.

Using flow cytometry, we identified and isolated a small numbers of SP cells, which are characterized by their ability to exclude Hoechst 33342 by ABC transporters. These SP cells have been shown to possess stem-cell characteristics [24]. We demonstrated an increased expression of stemness genes, including Nanog, *β*-catenin, Sox2, and CD133, all of which have been implicated and used in the identification and characterization of CSCs [25–27]. Although aberrant *β*-catenin activation has been prominently indicated for colorectal carcinogenesis and progression [15, 19, 28], its role in lung cancer and the generation of lung CSCs has not been well described. We observed that isolated A549 SP cells exhibited an elevated level of *β*-catenin activity and higher ability to form tumor spheroids. The ability to form tumor spheroids not only marks the self-renewal ability of these SP cells, but also represents an operational measure of the number of cancer-initiating cells within a tumor population [29]. Thus, the increased ability of A549 SP cells to form tumor spheroids strongly suggested that these cells resemble CSCs. More importantly, the correlation between high tumor spheroid-forming ability and increased *β*-catenin activity in A549 SP cells indicated that the *β*-catenin pathway could play an important role in the generation and maintenance of lung CSCs. Our data were further supported by studies where increased *β*-catenin signaling was implicated in cell fate determination of upper airway progenitor cells and the acceleration of lung cancer progression [18, 30–32]. 

Using high throughput platforms, different groups have been able to identify an antibiotic, Salinomycin, as an anticancer stem cell agent [33–35]. These studies provided an important precedent for the rapid identification of effective agents against CSCs. In this study, using CMAP, we identified another antibiotic, AMA, as a potential anti-CSC compound. AMA is an antifungal agent, that is, derived from *Streptomyces kitazawensis* [36] and is a potent inhibitor of succinate oxidase, NADH oxidase, and mitochondrial electron transport between cytochromes b and c [14, 37]. In addition, previous studies indicated that AMA induced apoptosis in lung cancer cells via the generation of reactive oxygen species and negative modulation of the MAPK signaling pathways [38, 39]. After establishing A549 SP cells as a representative CSC model, we tested AMA's ability to eliminate CSCs. Tumor spheroids from A549 SP cells appeared to be sensitive to AMA in a dose-dependent fashion, evident from the number of spheroids decreased as AMA concentration increased. AMA-treated tumor spheroids demonstrated suppressed expression levels of *β*-catenin and its signaling components including TCF-4, NF-*κ*B, and cyclin D1, which provides a partial explanation of how AMA was able to inhibit tumor spheroid formation. Therefore, the observation that AMA suppressed the formation of A549 tumor spheroids and downregulated the *β*-catenin signaling cascade suggests that it could be a potential anti-CSC agent.

One of the major obstacles in treating lung cancer patients is drug resistance. Patients who originally were responsive towards targeted therapeutic agents such as the EGFR inhibitors gefitinib and erlotinib inevitably develop resistance. In this study, we used the gefitinib-resistant lung cancer cell line PC-9/GR to demonstrate that under the presence of AMA, PC-9/GR cells became sensitive towards gefitinib. In addition, AMA and gefitinib were shown to synergistically suppress PC-9/GR SP cells. Another gefitinib-resistant NSCLC cell line H1975 (bearing T790 M mutation) was shown to be sensitive towards AMA treatment (Supplementary Figure  1), suggesting that AMA could target lung CSCs regardless of its EGFR genotype. Furthermore, the combination of AMA and gefitinib suppressed the expression of components of the *β*-catenin cascade. This observation is important since lung basal cell-specific *β*-catenin activation leads to increased cell proliferation, enhanced self-renewal ability, and induction of early components of early epithelial-mesenchymal transition (EMT), including increased Snail transcription and reduced E-cadherin expression [18]. 

Equally important, it has been suggested that cross-talk between signaling pathways may contribute to the cellular diversity associated with stem cells during embryogenesis and tissue maintenance and may have a key role in the generation/maintenance of CSCs. For instance, cross-talk was identified between the *β*-catenin and EGFR signaling cascades [40]. It is plausible that AMA sensitized PC-9/GR cells by suppressing *β*-catenin cascades, which led to the inhibition of EGFR signaling, thereby achieving dual inhibition of both signaling cascades (Supplementary Figure  2). As precisely how AMA affects EGFR signaling is not clear at the moment and is under our investigation. 

Of interest, our hypothesis and data were echoed and supported by recent studies in which various compounds that selectively target CSCs have been discovered. These agents include microbial-derived and plant-derived molecules that target key signaling pathways of CSCs [41, 42] or antibodies against CSC-specific cell surface markers [43]. Salinomycin, another antibiotic discovered using high throughput screening methodology, was shown to selectively kill human breast CSCs [33]. In that study, CD44^+^ CD24^−^ ALDH1^+^ breast CSCs treated with salinomycin alone demonstrated reduced colony forming efficiency and increased apoptosis; these effects were greater when salinomycin was combined with doxorubicin. Similar results were observed in our study, where gefitinib-resistant lung cancer cells (PC-9/GR) were more effectively killed by the combination of AMA and gefitinib than by AMA or gefitinib alone, providing evidence that AMA alone and/or in combination with targeted anticancer drugs effectively eliminate CSCs. In addition, both AMA (Figure 5) and salinomycin [33] promote the differentiation of cells. Notably, CSCs have been shown to contain lower ROS levels and are associated with increased expression of free radical scavenging systems [44]. Thus, pharmacological depletion of ROS scavengers could decrease their clonogenicity and resensitization towards pharmacological agents or radiation. Because AMA is a potent mitochondrial inhibitor [14, 37], the addition of AMA may decrease the ability of CSCs to combat treatment-induced oxidative stress and lead to their eradication. In support, AMA has been demonstrated to cause the loss of mitochondrial membrane potential in Calu-6 lung cancer cells. Interestingly, the intracellular ROS level was decreased in AMA-treated Calu-6 cells while O_2_^•^^−^ among ROS was increased. More importantly, AMA treatment induced GSH depletion in Calu-6 cells rendering the cells more susceptible to chemotherapeutic agents [39]. It is interesting to note that *β*-catenin signaling has been shown to activate ROS-defending enzymes such as GST1 (glutathione S transferase 1), which plays an important role in combating drug-induced ROS in the liver [45]. The observation where AMA treatment reduced *β*-catenin signaling provides additional explanation why AMA functioned synergistically with gefitinib and overcame gefitinib resistance in PC-9/GR cells. 

Our data and others suggest a possible link between dysfunctional mitochondrial activity and decreased self-renewal capacity by AMA treatment; this could be due to the decreased expression of *β*-catenin. We previously showed that *β*-catenin silencing led to the decreased expression of glutathione S-transferase pi 1 (GSTP1) and intracellular GSH level [46]. We believe that AMA suppresses the self-renewal ability of lung CSCs via multiple pathways including ROS modulation and more importantly decreased *β*-catenin signaling components (cyclin D1) as well as other stemness genes such as Sox2, Nanog, and CD133. Therefore, AMA (or other mitochondrial modulators) could be used in combination with currently available chemotherapeutic agents to eliminate CSCs and reduce the chance of recurrence. 

In conclusion, using CMAP, we were able to rapidly identify AMA as an antilung CSC agent. We provided evidence that AMA suppressed the self-renewal ability of lung CSCs through negative modulation of the *β*-catenin signaling cascade. More importantly, AMA in combination with gefitinib decreased the percentage of gefitinib-resistant PC-9/GR SP cells. Therefore, AMA could be used in combination with gefitinib to combat drug resistance in lung cancer patients. 

## Supplementary Material

Supplementary Table 1: A list of potential antibiotics which have the ability to reverse ESC signatures were calculated and obtained from Wong et al.
Supplementary Figure 1: Antimycin A treatment suppresses gefitinib-resistant H1970 NSCLC cells. H1975 NSCLC, harboring T790M mutation in the EGFR which renders gefitinib ineffective, appeared to be sensitive to AMA treatment.
Supplementary Figure 2: Antimycin A treatment leads to the suppression of EGFR expression and downstream AKT activity.

## References

[1] Jemal A, Siegel R, Ward E, Murray T, Xu J, Thun MJ (2007). Cancer statistics, 2007. *CA: A Cancer Journal for Clinicians*.

[2] Pao W, Chmielecki J (2010). Rational, biologically based treatment of *EGFR*-mutant non-small-cell lung cancer. *Nature Reviews Cancer*.

[3] Nguewa PA, Calvo A, Pullamsetti SS, Banat GA, Grimminger F, Savai R (2011). Tyrosine kinase inhibitors with antiangiogenic properties for the treatment of non-small cell lung cancer. *Expert Opinion on Investigational Drugs*.

[4] Maemondo M, Inoue A, Kobayashi K (2010). Gefitinib or chemotherapy for non-small-cell lung cancer with mutated *EGFR*. *The New England Journal of Medicine*.

[5] Mok TS, Wu YL, Thongprasert S (2009). Gefitinib or carboplatin-paclitaxel in pulmonary adenocarcinoma. *The New England Journal of Medicine*.

[6] Krause DS, van Etten RA (2007). Right on target: eradicating leukemic stem cells. *Trends in Molecular Medicine*.

[7] Liu JC, Deng T, Lehal RS, Kim J, Zacksenhaus E (2007). Identification of tumorsphere- and tumor-initiating cells in HER2/Neu-induced mammary tumors. *Cancer Research*.

[8] Ishii H, Iwatsuki M, Ieta K (2008). Cancer stem cells and chemoradiation resistance. *Cancer Science*.

[9] Yang YM, Chang JW (2008). Current status and issues in cancer stem cell study. *Cancer Investigation*.

[10] Chang A (2011). Chemotherapy, chemoresistance and the changing treatment landscape for NSCLC. *Lung Cancer*.

[11] Yang WL, Lee YE, Chen MH, Chao KM, Huang CY (2012). *In-silico* drug screening and potential target identification for hepatocellular carcinoma using support vector machines based on drug screening result. *Gene*.

[12] Seipke RF, Barke J, Brearley C (2011). A single *Streptomyces* symbiont makes multiple antifungals to support the fungus farming ant *Acromyrmex octospinosus*. *PLoS ONE*.

[13] Shaham O, Slate NG, Goldberger O (2010). A plasma signature of human mitochondrial disease revealed through metabolic profiling of spent media from cultured muscle cells. *Proceedings of the National Academy of Sciences of the United States of America*.

[14] Han YH, Kim SH, Kim SZ, Park WH (2008). Antimycin A as a mitochondrial electron transport inhibitor prevents the growth of human lung cancer A549 cells. *Oncology Reports*.

[15] Siemens H, Neumann J, Jackstadt R (2013). Detection of *miR-34a* promoter methylation in combination with elevated expression of c-Met and beta-catenin predicts distant metastasis of colon cancer. *Clinical Cancer Research*.

[16] Wong DJ, Liu H, Ridky TW, Cassarino D, Segal E, Chang HY (2008). Module map of stem cell genes guides creation of epithelial cancer stem cells. *Cell Stem Cell*.

[17] Kim J, Woo AJ, Chu J (2010). A Myc network accounts for similarities between embryonic stem and cancer cell transcription programs. *Cell*.

[18] Giangreco A, Lu L, Vickers C (2012). *β*-catenin determines upper airway progenitor cell fate and preinvasive squamous lung cancer progression by modulating epithelial-mesenchymal transition. *Journal of Pathology*.

[19] Han J, Sridevi P, Ramirez M, Ludwig KJ, Wang JY (2013). *β*-catenin-dependent lysosomal targeting of internalized tumor necrosis factor-*α* suppresses caspase-8 activation in apoptosis-resistant colon cancer cells. *Molecular Biology of the Cell*.

[20] Kim TM, Yim SH, Lee JS (2005). Genome-wide screening of genomic alterations and their clinicopathologic implications in non-small cell lung cancers. *Clinical Cancer Research*.

[21] Petty RD, Nicolson MC, Kerr KM, Collie-Duguid E, Murray GI (2004). Gene expression profiling in non-small cell lung cancer: from molecular mechanisms to clinical application. *Clinical Cancer Research*.

[22] Lin C, Song H, Huang C (2012). Alveolar type II cells possess the capability of initiating lung tumor development. *PLoS ONE*.

[23] Wang S, Xu ZY, Wang LF, Su W (2013). CD133+ cancer stem cells in lung cancer. *Frontiers in Bioscience*.

[24] Goodell MA, Brose K, Paradis G, Conner AS, Mulligan RC (1996). Isolation and functional properties of murine hematopoietic stem cells that are replicating *in vivo*. *Journal of Experimental Medicine*.

[25] Chiou SH, Wang ML, Chou YT (2010). Coexpression of *Oct4* and *Nanog* enhances malignancy in lung adenocarcinoma by inducing cancer stem cell-like properties and epithelial-mesenchymal transdifferentiation. *Cancer Research*.

[26] He A, Qi W, Huang Y (2012). CD133 expression predicts lung metastasis and poor prognosis in osteosarcoma patients: a clinical and experimental study. *Experimental and Therapeutic Medicine*.

[27] Singh S, Trevino J, Bora-Singhal N (2012). EGFR/Src/Akt signaling modulates Sox2 expression and self-renewal of stem-like side-population cells in non-small cell lung cancer. *Molecular Cancer*.

[28] Rosenbluh J, Nijhawan D, Cox AG (2012). *β*-catenin-driven cancers require a YAP1 transcriptional complex for survival and tumorigenesis. *Cell*.

[29] Fillmore CM, Kuperwasser C (2008). Human breast cancer cell lines contain stem-like cells that self-renew, give rise to phenotypically diverse progeny and survive chemotherapy. *Breast Cancer Research*.

[30] Pacheco-Pinedo EC, Durham AC, Stewart KM (2011). Wnt/*β*-catenin signaling accelerates mouse lung tumorigenesis by imposing an embryonic distal progenitor phenotype on lung epithelium. *Journal of Clinical Investigation*.

[31] Sidhu SS, Nawroth R, Retz M, Lemjabbar-Alaoui H, Dasari V, Basbaum C (2010). EMMPRIN regulates the canonical Wnt/*β*-catenin signaling pathway, a potential role in accelerating lung tumorigenesis. *Oncogene*.

[32] Yeh CT, Wu AT, Chang PM (2012). Trifluoperazine, an antipsychotic agent, inhibits cancer stem cell growth and overcomes drug resistance of lung cancer. *The American Journal of Respiratory and Critical Care Medicine*.

[33] Gupta PB, Onder TT, Jiang G (2009). Identification of selective inhibitors of cancer stem cells by high-throughput screening. *Cell*.

[34] Lu D, Choi MY, Yu J, Castro JE, Kipps TJ, Carson DA (2011). Salinomycin inhibits Wnt signaling and selectively induces apoptosis in chronic lymphocytic leukemia cells. *Proceedings of the National Academy of Sciences of the United States of America*.

[35] Rowan K (2009). High-throughput screening finds potential killer of cancer stem cells. *Journal of the National Cancer Institute*.

[36] Nakayama K, Okamoto F, Harada Y (1956). Antimycin A: isolation from a new *Streptomyces* and activity against rice plant blast fungi. *Journal of Antibiotics*.

[37] Han YW, Kim SZ, Kim SH, Park WH (2007). The changes of intracellular H_2_O_2_ are an important factor maintaining mitochondria membrane potential of antimycin A-treated As4.1 juxtaglomerular cells. *Biochemical Pharmacology*.

[38] Han YH, Moon HJ, You BR, Kim SZ, Kim SH, Park WH (2009). p38 inhibitor intensified cell death in antimycin A-treated As4.1 juxtaglomerular cells via the enhancement of GSH depletion. *Anticancer Research*.

[39] Han YH, Park WH (2010). The effects of MAPK inhibitors on antimycin A-treated Calu-6 lung cancer cells in relation to cell growth, reactive oxygen species, and glutathione. *Molecular and Cellular Biochemistry*.

[40] Latasa MU, Salis F, Urtasun R (2012). Regulation of amphiregulin gene expression by *β*-catenin signaling in human hepatocellular carcinoma cells: a novel crosstalk between FGF19 and the EGFR system. *PLoS ONE*.

[41] Kawasaki BT, Hurt EM, Mistree T, Farrar WL (2008). Targeting cancer stem cells with phytochemicals. *Molecular Interventions*.

[42] Li Y, Wicha MS, Schwartz SJ, Sun D (2011). Implications of cancer stem cell theory for cancer chemoprevention by natural dietary compounds. *Journal of Nutritional Biochemistry*.

[43] Ginestier C, Liu S, Diebel ME (2010). CXCR1 blockade selectively targets human breast cancer stem cells in vitro and in xenografts. *Journal of Clinical Investigation*.

[44] Diehn M, Cho RW, Lobo NA (2009). Association of reactive oxygen species levels and radioresistance in cancer stem cells. *Nature*.

[45] Giera S, Braeuning A, Köhle C (2010). Wnt/*β*-catenin signaling activates and determines hepatic zonal expression of glutathione S-transferases in mouse liver. *Toxicological Sciences*.

[46] Lin LC, Yeh CT, Kuo CC (2012). Sulforaphane potentiates the efficacy of imatinib against chronic leukemia cancer stem cells through enhanced abrogation of Wnt/*β*-catenin function. *Journal of Agricultural and Food Chemistry*.

