# Supplementary material for: A Preclinical Evaluation of Antimycin A as a Potential Antilung Cancer Stem Cell Agent
Source: Evid Based Complement Alternat Med. 2013 Jun 11;2013:910451. doi: 10.1155/2013/910451 (PMC3693105; doi:10.1155/2013/910451)
Supplement: Supplementary file 1 — Supplementary Table 1: A list of potential antibiotics which have the ability to reverse ESC signatures were calculated and obtained from Wong et al. Supplementary Figure 1: Antimycin A treatment suppresses gefitinib-resistant H1970 NSCLC cells. H1975 NSCLC, harboring T790M mutation in the EGFR which renders gefitinib ineffective, appeared to be sensitive to AMA treatment. Supplementary Figure 2: Antimycin A treatment leads to the suppression of EGFR expression and downstream AKT activity. [file 910451.f1.docx]

**Supplementary Materials.**

**For Supplementary Table 1.**

A list of potential antibiotics which have the ability to reverse ESC signatures were calculated and obtained from Wong et al. [16]. They are shown in Supplementary Table 1.

**Supplementary Table 1.** List of Antibiotics that could reverse ESC signatures from Wong et al.

| **Rank** | **Drug Name** | **Mean** | **N** | **Enrichment** | ***P*** |
| --- | --- | --- | --- | --- | --- |
| **17** | Antimycin A | -0.59 | 5 | -0.728 | 0.0032 |
| **65** | Sulconazole | -0.583 | 4 | -0.661 | 0.02988 |
| **74** | Thiostrepton | -0.539 | 4 | -0.638 | 0.04098 |

**For supplementary Figure 1.**

To demonstrate that AMA’s effect is not cell line specific, we obtained another non-small cell lung cancer cell line, H1975 which has been well characterized as gefitinib-resistant due to T790M mutation in the EGFR. H1975 cells were cultured in RPMI 1640 medium (GIBCO-Life Technologies, Inc., Gaithersburg, MD) supplemented with 10% FBS, penicillin (100UI/mL) and streptomycin (100UI/mL) at 37 ^o^C in humidified atmosphere with 5% CO_2_. Antimycin A was obtained from Antimycin A was dissolved in DMSO before diluting with growth medium to a final DMSO concentration of <0.05%. H1975 cells were seeded into 96-well plates in growth medium at 500 cells/well. After 24 h, the medium was replaced with fresh growth medium containing antimycin A, and the cells were incubated for another 48 h. The cells were then fixed by gently adding 50 𝜇L TCA (50%) to each well for a final TCA concentration of 10%, with subsequent incubation for 1 h at 4∘C. The plates were then washed 5 times with tap water and air dried. The dried plates were stained with 100 𝜇L of 0.4% (w/v) SRB prepared in 1% (v/v) acetic acid for 10min at room temperature. The plates were rinsed quickly 3 times with 1% acetic acid to remove unbound dye and then air dried. The bound dye was solubilized in 20mmol/L Tris base (100𝜇L/well) for 5 min on a shaker. Optical densities were read on a microplate reader (Molecular Devices, Sunnyvale, CA) at 562 nm.

It was shown that AMA suppressed the cell viability of H1975 cells.

**Legend for Supplementary Figure 1.** **Antimycin A treatment suppresses gefitinib-resistant H1970 NSCLC cells.** H1975 NSCLC, harboring T790M mutation in the EGFR which renders gefitinib ineffective, appeared to be sensitive to AMA treatment.

**For Supplementary Figure 2.**

To further explore the function of AMA in suppressing gefitinib-resistant lung cancer cells and lung CSCs, we decided to examine if AMA affects the expression of EGFR. A549 cells were subjected to AMA treatment as described in the Materials and Methods section and the expression level of EGFR and phosphorylated AKT was analyzed.

**Legend for Supplementary Figure 2. Antimycin A treatment leads to the suppression of EGFR expression and downstream AKT activity.** AMA treatment (48 h) in A549 NSCLC cells led to the decreased the expression level of EGFR and phosphorylated AKT in a dose-dependent manner. However, the exact underlying mechanism for AMA-mediated EGFR suppression is currently not clear. It is plausible that EGFR suppression was associated with the down-regulation of β-catenin (please refer to the discussion section).

Supplementary Figure 1.


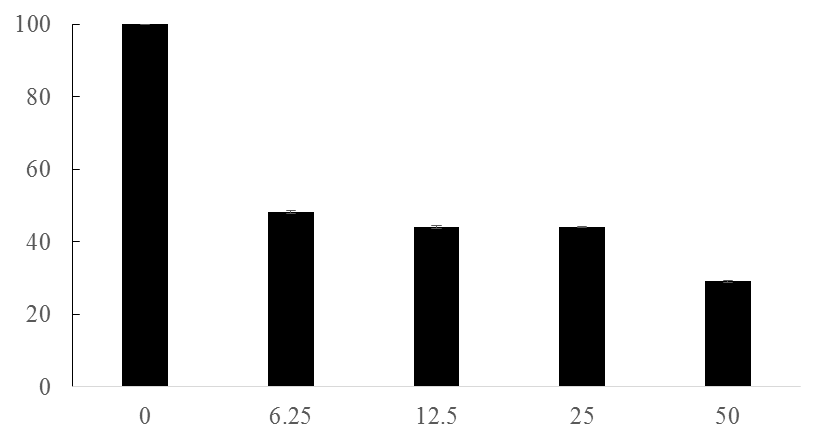


AMA (µM)

Cell Viability (% of control)

Supplementary Figure 2


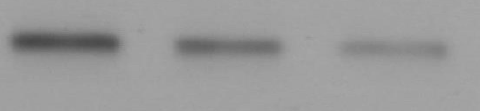

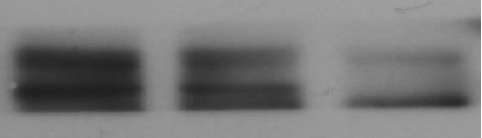

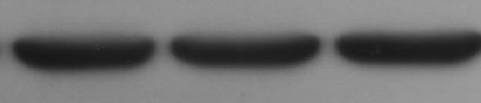


0 5 10

(µM)

EGFR

P-Akt

β-Actin
